# Department of Canine, Feline, and Avian Medicine and Surgery

**Published:** 1901-06

**Authors:** Cecil French

**Affiliations:** Washington, D. C.


### DEPARTMENT OF CANINE, FELINE, AND AVIAN MEDICINE AND SURGERY.

By Cecil French, D.V.S.,
WASHINGTON, D. C.

---

Experimental Rectal Injection in the Dog. Of recent date I have made certain experimental observations on seven dogs of both sexes and different ages and sizes, in order to watch the local effect produced by forcible injections of varying quantities of fluid into the bowel, and to see whether it agreed with that witnessed in cats by Senn.

Senn (*Intestinal Surgery*) experimented on three cats with the object of studying the effect of forcible enemata. The animals were etherized, the abdomen opened, and an incision made in the ileum just above the ileocæcal valve. Water was injected *per rectum*, and as the cæcum became distended it could readily be seen that the valve became tense and effectually prevented the escape of even a drop of the fluid into the ileum. The competency of the valve was only overcome by overdistention of the cæcum, which mechanically separated its margins and thereby allowed a fine stream of water to escape into the ileum. It was observed that multiple lacerations of the peritoneal surface of the gut took place as a result of the pressure. One of the animals sickened immediately after the experiment was made, and died eight days later from the effects of the injury received.

All of the animals (dogs) in my experiments were submitted to morphine-chloroform narcosis, and in no instance were they allowed to recover consciousness, but were allowed to die by opening bloodvessels.

An "alpha" bulb-syringe was used to make the injections, my object being to avoid using a degree of force in excess of that employed in everyday practice. The fluid used was cold water at a temperature of 51° F.

It was found somewhat difficult to make accurate computations of the amount of fluid which actually was thrown into the bowel, owing to the fact that it tended to be expelled past the nozzle of the syringe, not by any peristaltic action on the part of the bowel, for that was totally inhibited by the action of the drugs, but by the elasticity of the rectum coming into play as soon as the latter was distended. Fecal contents also offered a certain degree of obstruction, though the water in most cases broke these down and rendered them fluid.

Before any water was injected the abdominal cavity was widely opened and the whole of the gastro-intestinal viscera drawn outside the wound as much as possible, so that every portion might be under observation. The water, on being injected, advanced in a column, its progress keeping time to the impulse of each fresh influx conveyed by compressing the bulb, the bowel immediately in front of the advancing column being contracted and able to hold it back in check.

In every case, with a single exception, the ileocæcal valve proved to be patent and offered no obstruction whatever to the onflow of the water. In the exceptional case (of a male) the valve effectually prevented the passage of the water and it was impossible to overcome it by increased pumping. Neither did the wall of the rectum or colon suffer from the forcible distention to which they were thereby subjected.

In a fox-terrier the head of the column of water reached and passed the ileocæcal valve by the time one-half of a pint had been injected. In a toy-terrier one-quarter of a pint sufficed to produce the same effect. In a large hound it took less than a pint. In the fox-terrier one-half of a gallon was sufficient to traverse the whole extent of the intestines and reach the stomach, from which it escaped by the œsophagus and mouth when the head was lowered. In a setter it required over a gallon to effect passage of the entire tract. The cold water had a marked chilling effect on the bowel. In one anmial, whose bowel was not altogether withdrawn, the effect was very noticeable to the hand introduced within the cavity. The conclusions arrived at respecting the dog were as follows:

The ileocæcal valve is permeable in most animals, and fluids, when beyond certain definite amounts are injected, pass to the small intestine, and if in sufficient quantity may traverse the entire tract and be ejected from the stomach.

When the valve is not patent, it is impossible to produce any damaging effect on the bowel by forcible injection with the ordinary bulb syringe, the fluid escaping at the anus past the nozzle.

The quantity of fluid sufficient to distend the rectum and colon varies between one-quarter of a pint in the smallest animals and one pint in the largest.

To produce evacuation of the lower bowel no more than the minimum quantity need be injected.

Medicated injections employed for their local effect on inflammatory conditions of the intestinal mucosa, as well as nutritive enemata, may be advantageously used in larger quantities and forced beyond the ileocæcal valve.

In view of Senn's (*Intestinal Surgery*) successful reductions of artificial invaginations of the colon by forcible injection of water, there would appear to be no reason why the same means should not be effective when employed to reduce recent intussusceptions of the small bowel.

Cold fluids should not be used unless with the object of lowering bodily temperature.

